# Damage Location and Quantification Indices of Shear Structures Based on Changes in the First Two or Three Natural Frequencies

**DOI:** 10.1155/2014/459097

**Published:** 2014-08-27

**Authors:** Hien HoThu, Akira Mita

**Affiliations:** Mita Laboratory, Department of System Design Engineering, Keio University, 3-14-1 Hiyoshi, Kohoku-Ku, Yokohama 223-8522, Japan

## Abstract

This study proposes damage detection algorithms for multistory shear structures that only need the first two or three natural frequencies. The methods are able to determine the location and severity of damage on the basis of damage location indices (DLI) and damage quantification indices (DQI) consisting of the changes in the first few squared natural frequencies of the undamaged and damaged states. The damage is assumed to be represented by a reduction in stiffness. This stiffness reduction causes a shift in the natural frequencies of the structure. The uncertainty associated with system identification methods for obtaining natural frequencies is also carefully considered. The methods are accurate and cost-effective means only requiring the changes in the natural frequencies.

## 1. Introduction

Structural health monitoring (SHM) systems are garnering attention as a way of maintaining building structures subject to natural hazards such as large earthquakes and strong winds [1]. SHM systems play an important role in assessing the health of a structure because they can determine the location and the severity of damage. The obstacle we often face when installing an SHM system in a building is the trade-off between the numbers of sensors and the accuracy of damage detection. A large number of sensors are costly and entail a large effort for wiring and installation. Such complicated and expensive SHM systems are not feasible for most buildings [2]. Therefore, a good SHM system should have few sensors yet obtaining enough information about the health of a structure.

Doebling et al. [3] graded SHM systems into four levels of ability, as follows:

- Level 1: determining that damage is present in the structure;
- Level 2: determination of the location of the damage;
- Level 3: quantification of the severity of the damage;
- Level 4: prediction of the remaining service life of the structure.

The first three levels are most often related to structural dynamic testing and modeling issues. Level 4 is not addressed in the structural vibration or modal analysis literature. Hence, most of damage detection methods aim to classify damage into the first three levels.

Damage detection algorithms based on the modal properties of a structure, such as modal frequencies, mode shapes, curvature mode shapes, and modal flexibilities, have been studied in the SHM field for decades. However, most algorithms have difficulties in identifying the precise location and magnitude of the damage. Their accuracy and reliability are not considered sufficient, if not completely inadequate [2]. The key to making a successful damage detection method is thus using a few modal properties of a structure to identify Levels 1, 2, and 3.

Zhao and DeWolf [4] presented a sensitivity study comparing the use of natural frequencies, mode shapes, and modal flexibilities for monitoring. Based on the fact that natural frequencies are sensitive indicators of structural integrity, the relationship between frequency changes and structural damage was discussed in a review by Salawu [5]. These studies showed that sensitivity analysis of the natural frequencies can be a valuable tool in SHM.

A damage detection method based on natural frequencies only needs two vibration sensors (or even one acceleration sensor) to obtain the modal frequencies. It is known that, of the various characteristics, the natural frequencies are the least contaminated by measurement noise and can generally be measured with good accuracy [6]. Messina et al. [7] suggested a standard error of 0.15% as a benchmark figure for natural frequencies measured in the laboratory with the impulse hammer technique. Some researches [8–14] have achieved a standard deviation of less than 1% for the first few modes of natural frequencies. This low level of error suggests that a damage detection method using only information on the natural frequencies would have acceptable accuracy.

Although many previous studies concluded that the frequency cannot provide spatial information about structural changes, multiple frequency shifts may provide spatial information about structural damage in situations where many natural frequencies can be measured [15]. However, there are only a few natural frequencies that can be measured in most buildings. Therefore, a natural frequency based method would need to work with only a few frequencies if it were to be practical.

The purpose of this study is to devise a damage detection method to identify the existence, location, and amount of damage to multistory shear structures by using new damage indices consisting of the changes in the first two or three natural frequencies.

## 2. Sensitivity of Squared Natural Frequency Changes to Structural Damage

**Figure 1:**
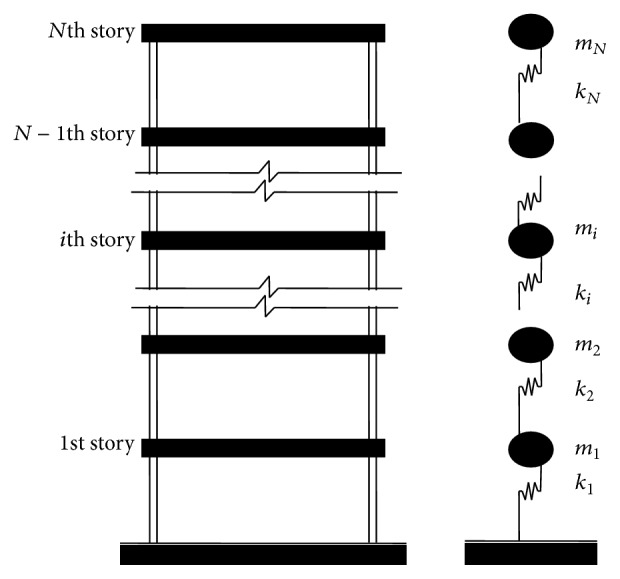
Simplified structural model with *N* degrees of freedom.

A multistory shear structure (*N*-story) can be modeled as a one-dimensional lumped mass shear model, as shown in Figure 1. Most of the damage to a structure, such as cracks, fatigue, corrosion, and loosening of bolted joints, manifests itself as a stiffness reduction.

The characteristic equation for such a structure is written as

(1) $$\begin{array}{c} \left[K-\omega_{r}^{2}M\right]\emptyset_{r}=0, \end{array}$$

where *ω*_*r*_ and **∅**_*r*_ are the *r*th frequency and mode shape vector, respectively,

**K** is the stiffness matrix (*N* × *N*),
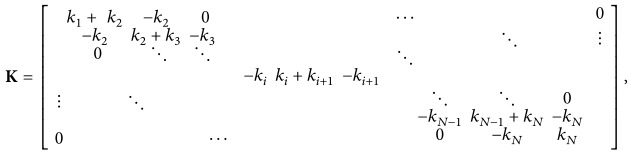
(2)and** M** is the mass matrix (*N* × *N*),

(3) M=[m10  0m2    ⋱…          0                          ⋱⋮                                      mi                                                      ⋮⋱            0⋯⋱mN−1    00      mN].

Solving (1) in the manner of (8) in [15], the general expression for the *i*th story stiffness can be obtained as

(4) $$\begin{array}{c} k_{i}=\frac{\omega_{r}^{2}}{\left(\phi_{ir}-\phi_{(i-1)r}\right)}\overset{N}{\underset{j=i}{\sum }}m_{j}\phi_{jr}, \end{array}$$

where *ϕ*_*ir*_ and *ϕ*_(*i*−1)*r*_ are the *r*th mode shapes at the *i*th and (*i* − 1)th stories, respectively.

In addition, the squared natural circular frequency of the *r*th mode is expressed in [2] as follows:

(5) $$\begin{array}{c} \omega_{r}^{2}=\frac{K_{r}}{M_{r}}=\frac{\phi_{r}^{T}K\phi_{r}}{\phi_{r}^{T}M\phi_{r}}, \end{array}$$

where *ω*_*r*_ is the *r*th natural circular frequency, *K*_*r*_ is the *r*th modal stiffness, *M*_*r*_ is the *r*th modal mass, **K** and **M** are the stiffness and mass matrices, and **ϕ**_*r*_ is the *r*th mode shape vector.

Although the yielded structure is undamaged and may also have reduced stiffness sometime later, in this study, we will assume that the structural damage can be directly expressed as a stiffness reduction and that the structural mass remains unchanged. The difference between the squared natural frequencies of the *r*th mode of the undamaged structure and the same structure with damage to the *i*th story is expressed as

(6) $$\begin{array}{c} \omega_{r}^{2}-\omega_{r\left(i\right)}^{2}=\frac{\phi_{r}^{T}K\phi_{r}}{\phi_{r}^{T}M\phi_{r}}-\frac{\phi_{r(i)}^{T}K_{i}\phi_{r(i)}}{\phi_{r(i)}^{T}M\phi_{r(i)}}, \end{array}$$

where the subscript (*i*) denotes the *i*th damaged story state.

Here, *ω*_*r*_ and *ω*_*r*(*i*)_ are the *r*th frequencies of the undamaged and *i*th story damaged state, **K**_*i*_ is the global stiffness matrix with the stiffness reduction considered to be a reflection of damage to the *i*th story, and is the *r*th mode shape vector of the *i*th damaged story state.

In [16], Morita et al. gave an equation to obtain the difference in the squared natural frequency of the *r*th mode in (6) by neglecting the difference in mode shape arising from damage. That difference becomes

(7) $$\begin{array}{c} \omega_{r}^{2}-\omega_{r\left(i\right)}^{2}=\frac{\phi_{r}^{T}{\Delta K}_{i}\phi_{r}}{\phi_{r}^{T}M\phi_{r}}, \end{array}$$

where Δ**K**_*i*_ is the change in the stiffness matrix with the *i*th damaged story. When the *i*th story stiffness is reduced, only *k*_(*i*−1)(*i*−1)_, *k*_(*i*−1)*i*_, *k*_*i*(*i*−1)_ and *k*_*ii*_ are altered in the stiffness matrix. Δ**K**_*i*_ is written as

(8) $$\begin{array}{c} \Delta K_{i}=\left[\begin{array}{ccc} 0 & \ldots & 0\\ \vdots & \begin{array}{cc} k_{\left(i-1)(i-1\right)} & k_{(i-1)i}\\ k_{i(i-1)} & k_{ii} \end{array} & \vdots \\ 0 & \ldots & 0 \end{array}\right] \end{array}$$

or

(9) $$\begin{array}{c} \Delta K_{i}=\left[\begin{array}{ccc} 0 & \ldots & 0\\ \vdots & \begin{array}{cc} {\Delta k}_{i} & -{\Delta k}_{i}\\ -{\Delta k}_{i} & {\Delta k}_{i} \end{array} & \vdots \\ 0 & \ldots & 0 \end{array}\right], \end{array}$$

where Δ*k*_*i*_ is the change in stiffness of the *i*th story.

The squared natural frequency change ratio is determined by dividing both sides of (7) by *ω*_*r*_^2^:

(10) $$\begin{array}{c} \Delta_{i}^{\left(r\right)}=\frac{\omega_{r}^{2}-\omega_{r\left(i\right)}^{2}}{\omega_{r}^{2}}=\frac{\phi_{r}^{T}{\Delta K}_{i}\phi_{r}}{\omega_{r}^{2}\phi_{r}^{T}M\phi_{r}}=\;\frac{\phi_{r}^{T}{\Delta K}_{i}\phi_{r}}{\phi_{r}^{T}K\phi_{r}} \end{array}$$

(11) $$\begin{array}{c} ⟺\Delta_{i}^{\left(r\right)}=\frac{1}{\phi_{r}^{T}K\phi_{r}}\Delta k_{i}{\left(\phi_{ir}-\phi_{(i-1)r}\right)}^{2}, \end{array}$$

where Δ*k*_*i*_ is the change in stiffness of the *i*th story and *ϕ*_*ir*_ and *ϕ*_(*i*−1)*r*_ are the *r*th mode shapes at the *i*th and (*i* − 1)th story.

From (11), Δ*k*_*i*_ is the extent of damage to the *i*th story and (*ϕ*_*ir*_−*ϕ*_(*i*−1)*r*_)^2^ depends on the location of the *i*th damaged story. As damage normally causes a decrease in the natural frequencies, Δ_*i*_^(*r*)^ is positive when damage occurs. This frequency change ratio Δ_*i*_^(*r*)^ may vary depending on the damage location and quantification. Note that, for each natural frequency, there are some locations where the frequency is most sensitive to the damage, while there are other locations where the damage has little influence on the frequency.

Similarly, the frequency change ratio of the *s*th mode, Δ_*i*_^(*s*)^, is obtained as

(12) $$\begin{array}{c} \Delta_{i}^{\left(s\right)}=\frac{1}{\phi_{s}^{T}K\phi_{s}}\Delta k_{i}{\left(\phi_{is}-\phi_{(i-1)s}\right)}^{2}. \end{array}$$

The sum of these changes is

(13) Δi(r)+Δi(s)=Δki[1ϕrTKϕr(ϕir−ϕ(i−1)r)20000000+1ϕsTKϕs(ϕis−ϕ(i−1)s)2].

Thus, the ratio of the changes in the two natural frequencies of the *r*th and *s*th modes can be written as

(14) Δi(r)Δi(r)+Δi(s)=(1/ϕrTKϕr)(ϕir−ϕ(i−1)r)2(1/ϕrTKϕr)(ϕir−ϕ(i−1)r)2+(1/ϕsTKϕs)(ϕis−ϕ(i−1)s)2.

(15) ⟹Δi(r)Δi(r)+Δi(s)=11+(ϕrTKϕr/ϕsTKϕs)((ϕis−ϕ(i−1)s)/(ϕir−ϕ(i−1)r))2

From (4), we can use the *r*th mode or *s*th mode to obtain the stiffness of the *i*th story:

(16) $$\begin{array}{c} \frac{\omega_{r}^{2}}{\left(\emptyset_{ir}-\emptyset_{(i-1)r}\right)}\overset{N}{\underset{j=i}{\sum }}m_{j}\emptyset_{jr}=\frac{\omega_{s}^{2}}{\left(\emptyset_{is}-\emptyset_{(i-1)s}\right)}\overset{N}{\underset{j=i}{\sum }}m_{j}\emptyset_{js} \end{array}$$

(17) $$\begin{array}{c} ⟹\frac{\phi_{is}-\phi_{(i-1)s}}{\phi_{ir}-\phi_{(i-1)r}}=\frac{\omega_{s}^{2}}{\omega_{r}^{2}}\frac{\sum_{j=i}^{N}m_{j}\emptyset_{js}}{\sum_{j=i}^{N}m_{j}\emptyset_{jr}}. \end{array}$$

Substituting (17) into (15), we get

(18) Δi(r)Δi(r)+Δi(s)=11+(ϕrTKϕr/ϕsTKϕs)(ωs4/ωr4)(∑j=iNmj∅js/∑j=iNmj∅jr)2.

This ratio depends on the location of damage *i* and the number of degrees of freedom, in the following coefficient: (∑_*j*=*i*_^*N*^ *m*_*j*_ *∅*_*js*_/∑_*j*=*i*_^*N*^ *m*_*j*_ *∅*_*jr*_) = (*m*_*i*_ *∅*_*is*_ + *m*_*i*+1_ *∅*_(*i*+1)*s*_ + ⋯+  *m*_*N*_ *∅*_*Ns*_)/(*m*_*i*_ *∅*_*ir*_ + *m*_*i*+1_ *∅*_(*i*+1)*r*_ + ⋯+  *m*_*N*_ *∅*_*Nr*_) for *i* = 1, 2,…, *N*.

## 3. Proposed Approach to Damage Detection

Let us formulate a simple algorithm for detecting structural damage, its location, and its amount. Note that the existence of damage is determined by a change in the first two or three natural frequencies. In previous, some researches [8–14] have achieved a standard deviation of less than 1% for the first few modes of natural frequencies. In particular, we assume that the squared natural frequencies have a standard deviation of 1% and damage has occurred when the squared natural frequency changes by 2% or more.

### 3.1. Damage Location Indices

The previous section derived the ratio of the changes in two squared natural frequencies of the *r*th and *s*th modes. Like (18), this value depends on the location of the damage. The damage location index (DLI) is defined as

(19) $$\begin{array}{c} \text{DL}{\text{I}}^{rs}=\frac{\Delta_{i}^{\left(r\right)}}{\Delta_{i}^{\left(r\right)}+\Delta_{i}^{\left(s\right)}}. \end{array}$$

The number of obtained frequencies is usually smaller than the number of degrees of freedom *N*. When two of the first two or three frequencies are used, the DLI values are written as DLI^12^, DLI^23^, and so on:

(20) $$\begin{array}{c} {\text{DLI}}^{12}=\frac{\Delta_{i}^{\left(1\right)}}{\Delta_{i}^{\left(1\right)}+\Delta_{i}^{\left(2\right)}}\\ \text{DL}{\text{I}}^{23}=\frac{\Delta_{i}^{\left(2\right)}}{\Delta_{i}^{\left(2\right)}+\Delta_{i}^{\left(3\right)}}. \end{array}$$

The DLI^12^ value depends on the number of damaged stories *i*, and it can be used as a damage location indicator in the *N-*story shear structure. When taller building has many stories, DLI^23^ and/or DLI^34^ and so forth, can be used in addition to DLI^12^ to detect the damaged story.

### 3.2. Uncertainty of Damage Location Indices

The modal parameters are often sensitive to various environmental conditions such as temperature, humidity, and excitation amplitude. The effect of environmental conditions or excitation amplitude is treated as “noise” in a simulation, so we should obtain a confidence interval on the modal parameters.

Denoting by *σ*_*ω*^2^_ the standard deviation of the squared natural frequency, we will discuss the reliability of our method on the basis of the theory presented in [17]. We will assume that the squared natural frequencies have a standard deviation of 1%.

The standard deviation of the difference between the squared natural frequencies is

(21) $$\begin{array}{c} \sigma_{\left(\omega_{r}^{2}-\omega_{r(i)}^{2}\right)}=\sqrt{{\left(\sigma_{\omega_{r}^{2}}\right)}^{2}+{\left(\sigma_{\omega_{r(i)}^{2}}\right)}^{2}}. \end{array}$$

Moreover, the standard deviation of the changes in the *r*th frequency Δ^(*r*)^ is given by

(22) $$\begin{array}{c} \sigma_{\Delta^{\left(r\right)}}=\Delta^{\left(r\right)}\sqrt{{\left(\frac{\sigma_{\left(\omega_{r}^{2}-\omega_{r(i)}^{2}\right)}}{\omega_{r}^{2}-\omega_{r\left(i\right)}^{2}}\right)}^{2}+{\left(\frac{\sigma_{\omega_{r}^{2}}}{\omega_{r}^{2}}\right)}^{2}}. \end{array}$$

The standard deviation of the changes in the *s*th frequency *σ*_Δ^(*s*)^_ is calculated similarly to *σ*_Δ^(*r*)^_ in (22).

The standard deviation of the sum of the changes in the first two frequencies is

(23) $$\begin{array}{c} \sigma_{\left(\Delta^{\left(r\right)}+\Delta^{\left(s\right)}\right)}=\sqrt{{\left(\sigma_{\Delta^{\left(r\right)}}\right)}^{2}+{\left(\sigma_{\Delta^{\left(s\right)}}\right)}^{2}}. \end{array}$$

The standard deviation of the DLI can be calculated as

(24) $$\begin{array}{c} \sigma_{\text{DLI}\;}=\text{DLI}\;\sqrt{{\left(\frac{\sigma_{\Delta^{\left(r\right)}}}{\Delta^{\left(r\right)}}\right)}^{2}+{\left(\frac{\sigma_{\left(\Delta^{\left(r\right)}+\Delta^{\left(s\right)}\right)}}{\Delta^{\left(r\right)}+\Delta^{\left(s\right)}}\right)}^{2}}. \end{array}$$

Statistically, we expect the DLI to be reliable within 〈DLI ± 2*σ*_DLI_〉. Hence, the confidence interval is 95%, and the DLI can identify the damage location in a shear structure with satisfactory reliability.

### 3.3. Damage Quantification Indices

Now let us formulate a simple damage quantification technique. The change in the natural frequency can be used to interpolate the amount of damage at the detected location [18]. Equation (11) also shows that the change in the natural frequency is proportional to the stiffness reduction. In addition, Zhao and DeWolf [4] said that the change in natural frequency has a different sensitivity level for each location. That is, some stories show less of change in the natural frequency when the damage occurs. As [18], we can use the first frequency change Δ^(1)^ to interpolate the extent of damage. However, these values are too small for some free-end stories that are less sensitive to damage. Similarly, the second frequency change Δ^(2)^ also has less sensitive locations. In these cases, it is difficult to interpolate the extent of damage from the change in only one frequency.

The DLI defined in Section 3.1 was used to detect the location of a damaged story in the shear structure. Normally, DLI^12^ is composed of the changes in the first two or three natural frequencies. Equation (7) in [16] can be used to approximate the extent of damage from the change in any one of the obtained frequencies. A more reliable value can be found by averaging the estimates of all of the modes used in the analysis.

Because the sensitivity to a stiffness reduction depends on the modes, it would be a good idea to introduce the following averaging method. The damage quantification index (DQI) is defined by the changes in the first two or three frequencies that were used to determine the damaged story:

(25) $$\begin{array}{c} \text{DQI}=\frac{\sum_{j=1}^{2(3)}w_{j}{\Delta k}_{i}^{\left(j\right)}}{\sum_{j=1}^{2(3)}w_{j}}=\frac{w_{1}{\Delta k}_{i}^{\left(1\right)}+w_{2}{\Delta k}_{i}^{\left(2\right)}\left(+w_{3}{\Delta k}_{i}^{\left(3\right)}\right)}{w_{1}+w_{2}\left(+w_{3}\right)}, \end{array}$$

where Δ*k*_*i*_^(*j*)^ is the stiffness reduction of the damaged story *i*th, interpolated by the change in square frequency of the *j*th mode (Δ_*i*_^(*j*)^), and *w*_*j*_ is a weight with the variance, given by *w*_*j*_ = 1/(*σ*_Δ^(*j*)^_)^2^.

The reliability band of Δ*k*_*i*_^(*j*)^ is obtained from *σ*_Δ_*i*_^(*j*)^_ in (22) and the standard deviation of DQI, (*σ*_DQI  _), is also calculated.

## 4. Performance of Proposed Method

A four-story shear structure was measured to show the feasibility of the proposed method. It was modeled as a one-dimensional lumped mass shear model, as shown in Figure 1. The damping ratio for each mode was chosen to be 3%. The sampling frequency 200 Hz is commonly used in major monitoring systems for real buildings. However, as we can only need lower natural frequencies, the sampling frequency can be as low as 50 Hz provided that an appropriate antialiasing filter is used. In this study, the data sampling frequency was chosen 200 Hz. The excitation loading is white-noise. Although we used white-noise in this paper, the excitation can be any, such as winds, microtremors, and earthquakes.

The stories of the structure had the same mass, *m*_*i*_ = 1000 tons, but their stiffness was different: *k*_1_ = *k* = 1.3 × 10^3^ MN/m, *k*_2_ = 0.9*k*, *k*_3_ = 0.8*k*, and *k*_4_ = 0.7*k*. The first two natural frequencies of the undamaged structure were 1.89 Hz and 5.19 Hz. Four cases of damage (damage to the 1st, 2nd, 3rd, and 4th stories) were studied. The damage was simulated by reducing the stiffness of each story by 5%, 10%, 15%, 20%, 25%, and 30%.

**Figure 2:**
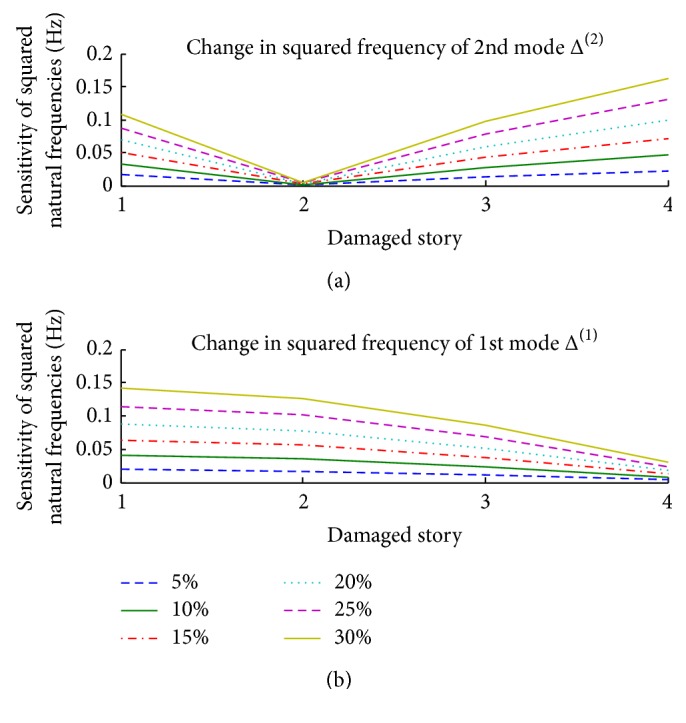
Sensitivity to changes in square natural frequencies (Δ^(1)^ and Δ^(2)^) of 4-story structure.

Figure 2 shows the sensitivity of the changes in the first two natural frequencies to the location of the damaged story. From Figure 2, it can be seen that the influence of the damage location identified by the first natural frequency decreases on higher stories; the results mean that the first natural frequency is the most sensitive to damage on the 1st story. By contrast, the second natural frequency is the least sensitive to damage on the 2nd story.

**Figure 3:**
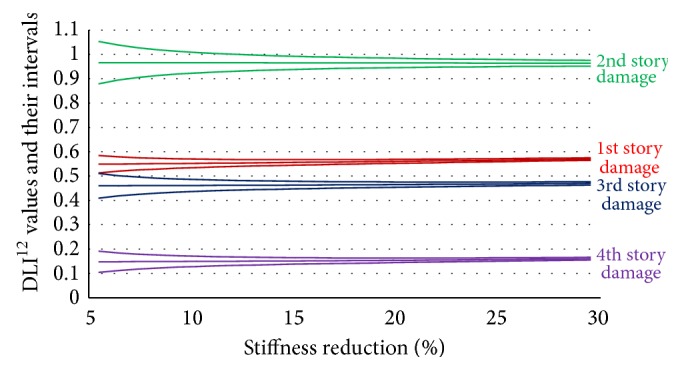
DLI^12^ values and their intervals within the range 〈DLI ± 2*σ*_DLI_〉 of 4-story structure.

Figure 3 uses different colors to plot DLI^12^ values and their confidence interval of 5% to 30% stiffness reduction for each story. The DLI^12^ values of these cases are indicated by the bold lines in the middle of each color; their reliability band considering 2*σ*_DLI_ is indicated by the two thin lines above and below each thick line (the standard deviation of the squared natural frequencies is assumed to be 1%).

From Figure 3, we can see the following.

(i) The DLI^12^ values are stable at each damaged story for any level of damage; the location of the damaged story can be detected by examining the correlation between the unknown data and the data of the story on which damage was detected.
(ii) The variances of 2*σ*_DLI_ depend on the stiffness reduction; when the reduction in stiffness is smaller, these values become larger. So the extent of damage, as indicated by the stiffness reduction, must be big enough to detect the location of damage without any mistakes.

**Figure 4:**
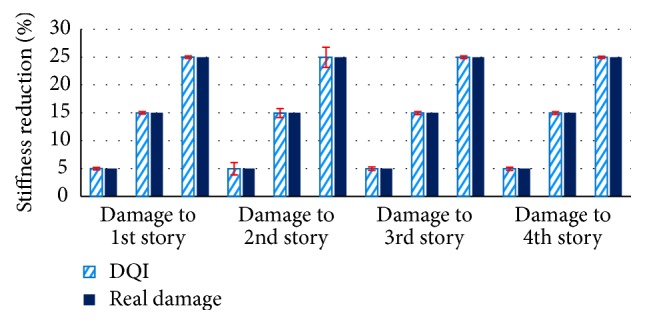
Comparison of DQI with uncertainty and actual damage of the 4-story structure.

In this simulation, two modes were enough to detect the damaged story. After determining the location of the damage by using the DLI values presented in Figure 3, the quantification of damage on that story can be calculated from the DQI definition with its standard deviation.  Figure 4 compares the DQI with its uncertainty against real damage to a 4-story structure that reduced the stiffness of each story by 5, 15, and 25%.

## 5. Application to Tall Buildings

Next, we verified our method on an eight-story structure. The damping ratio was chosen to be 3% and the data sampling frequency was 200 Hz; the excitation loading is white-noise. The mass of each story was 1000 tons. The stiffness of the first story was assumed to be *k*_1_ = *k* = 1.3 × 10^3^ MN/m, and the stiffness of the other stories was *k*_2_ = 0.95*k*, *k*_3_ = 0.9*k*, *k*_4_ = 0.85*k*, *k*_5_ = 0.8*k*, *k*_6_ = 0.75*k*, *k*_7_ = 0.7*k*, and *k*_8_ = 0.65*k*.

The first three natural frequencies of the undamaged structure were calculated to be 0.99, 2.82, and 4.58 Hz.  Table 1 summarizes these three cases in this simulation. The first three natural frequencies were obtained from acceleration data of the sensor on top of the simulated structure by applying the subspace identification method described in [19]. The resulting DLI values are displayed in Table 2.

**Figure 5:**
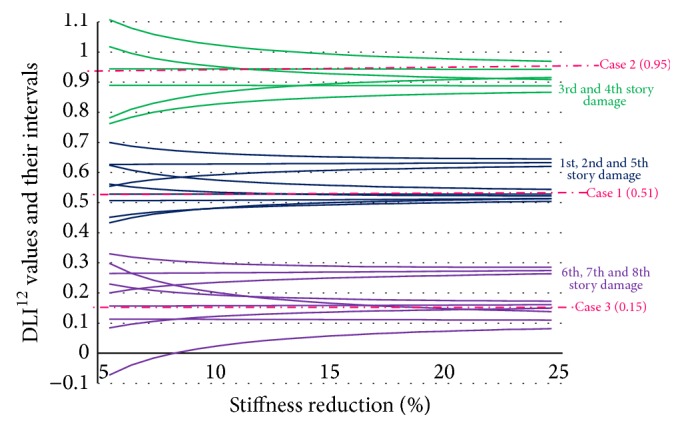
DLI^12^ values and their intervals for 8-story structure.

Eight cases of damage (from the 1st to 8th story) were simulated. For each case, the damage was simulated by reducing the stiffness of each story from 5 to 25%, and these 21 levels of damage were reflected in the changes in the natural frequencies. The DLI^12^ values and their reliability band were calculated by assuming that the squared frequency had a standard deviation of 1% (Figure 5). There is some overlap of the 95% confidence intervals. The DLI^12^ values could detect the location of the damage in three sections of the structure. The DLI^12^ values of the three damage cases are plotted as the dash dotted lines in Figure 5.

**Figure 6:**
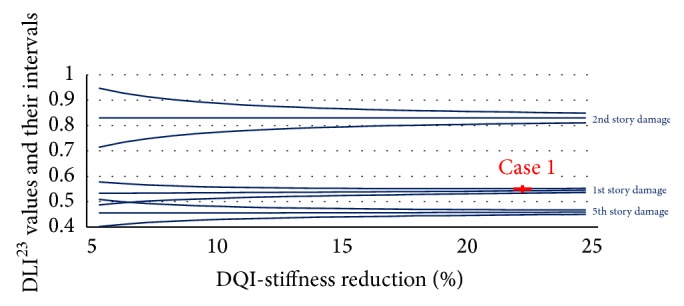
DLI^23^ and DQI values and their intervals for the blue sections.

**Figure 7:**
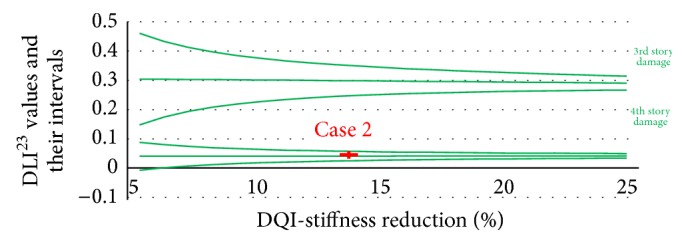
DLI^23^ and DQI values and their intervals for the green section.

**Figure 8:**
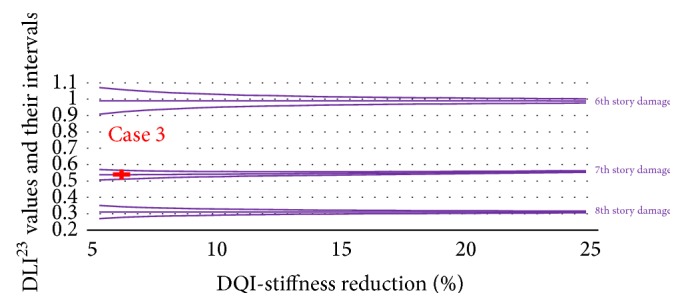
DLI^23^ and DQI values and their intervals for the purple section.

The next step is using the 2nd and 3rd frequency changes to get DLI^23^ and their reliability band. Figures 6, 7, and 8 plot the DLI^23^ values.

After determining the location of the damage by using the DLI values, the amount of damage on that story was calculated by interpolating the DQI values. The DLI and DQI values together with their uncertainty in the three damage cases are displayed as the red crosses in Figures 6 to 8.

Damage indices are dependent on the mass and stiffness distribution. Thus, we need a prior knowledge of them to apply our method. If design drawing is not available, some system identification tools may be needed. This is the limitation of this method to be applied to a real building.

## 6. Conclusion

The presented method determines the location and amount of damage to shear structures by using the changes in the first few natural frequencies only. Natural frequencies decrease as a result of damage and the damage indices (DLI and DQI) use these changes to reveal the location and amount of damage. The DLI uses two natural frequency changes between undamaged and damaged states and is stable for each damaged story. After identifying the location of the damage, the DQI of the damaged story is used to quantify the damage.

As we need only a few natural frequencies, two vibration sensors are enough to obtain the modal frequencies, one on the ground detecting an input and the other on the roof detecting an output. If the input lasts long and the spectrum is flat, we may identify those parameters using the output data without input information. Thus, in such a case, only one sensor is needed.

The uncertainty associated with system identification methods for obtaining natural frequencies was also carefully considered, and the confidence intervals of the DLI values were acceptable with high accuracy. The method was also shown to be able to detect the location of damage to tall buildings.

## Figures and Tables

**Table 1:** Damage scenarios of eight-story structure.

Damage case number	Location of damage (damaged story)	Quantification of damages (percentage of the stiffness reduction)
1	1st	23%
2	4th	14%
3	7th	6%

**Table 2:** Natural frequencies of 3 damage cases of eight-story structure.

Damage case number	The 1st natural frequency, *ω*_1_ [Hz]	The 2nd natural frequency, *ω*_2_ [Hz]	The 3rd natural frequency, *ω*_3_ [Hz]	DLI^12^	DLI^23^
1	0.97	2.75	4.47	0.51	0.52
2	0.98	2.82	4.50	0.95	0.04
3	0.99	2.76	4.49	0.15	0.55
